# Woody plant encroachment alters the vegetative and reproductive phenology of a *Vereda* palm swamp in Cerrado

**DOI:** 10.1007/s00484-026-03256-8

**Published:** 2026-06-30

**Authors:** Nathan Felipe Alves, João Paulo Costa, Patricia Morellato, Paulo Eugênio Oliveira

**Affiliations:** 1 Centro de Pesquisa em Dinâmica da Biodiversidade e Mudanças Climáticas e Departamento de Biodiversidade, Laboratório de Fenologia, UNESP - Universidade Estadual Paulista, Instituto de Biociências, Rio Claro, São Paulo Brazil; 2 Institute of Biology, Federal University of Uberlândia, Uberlândia, Minas Gerais Brazil

**Keywords:** Remote sensing, Wetlands, Biodiversity loss, Water loss, Ecosystem changes, Drones

## Abstract

Woody plant encroachment (WPE) is widespread in savanna and wetland ecosystems, yet its effects on phenological dynamics at fine spatial scales remain poorly quantified. Here, we assessed how woody plant encroachment alters vegetative and reproductive phenology in a *Vereda* palm swamp ecosystem of the Brazilian Cerrado by integrating high-resolution unmanned aerial vehicle (RPAS; Remotely Piloted Aircraft System) imagery with ground-based phenological monitoring. Ten regions of interest representing WPE and non-invaded native *Vereda* vegetation were monitored monthly for twelve consecutive months using drone imagery, with 1 × 1 m sampling grids (≈ 260 m² per region). Vegetation greenness was quantified from monthly orthomosaics using the Green Chromatic Coordinate (GCC) and analyzed with linear mixed-effects models including vegetation type, time, and their interaction. Flowering phenology was monitored biweekly from January to December 2023 in transects located in the middle region of the same *Vereda* area. Three WPE plant species and the native *Vereda* community occurring in the same area were analyzed using circular statistics and permutation tests. Woody plant encroachment areas exhibited significantly higher GCC values than native *Vereda* vegetation, indicating greater and more persistent greenness throughout the year. A significant vegetation type × time interaction revealed distinct phenological trajectories, with WPE areas maintaining elevated greenness during dry–wet seasonal transitions and possibly resulting in greater evapotranspiration and water use during the end of the dry season. Flowering phenology of WPE species was highly synchronized (mean angle = 3.45 rad; *r* = 0.65), whereas native *Vereda* species showed a more diffuse flowering pattern (mean angle = 1.99 rad; *r* = 0.27). Although a temporal flowering peak shift of approximately three months was observed between groups, this difference was not statistically significant. Overall, woody plant encroachment substantially modifies vegetative phenological dynamics and tends to homogenize flowering timing in *Vereda* ecosystems, highlighting the value of integrating RPAS-based monitoring and field observations to detect functional changes in Cerrado wetlands.

**Supplementary Information:**

The online version contains supplementary material available at 10.1007/s00484-026-03256-8.

## Introduction

The Cerrado is the most biodiverse tropical savanna in the world and the second largest biome in Brazil (Cardoso et al. 2026). This ecosystem plays a fundamental role in biodiversity conservation and in supporting human populations in the central region of the country, providing a wide range of ecosystem services, including carbon storage and water regulation for major South American river basins (Vieira et al. 2022). The Cerrado comprises a variety of vegetation types, among which the *Veredas* palm swamps stand out. These wetland ecosystems are legally protected and widely distributed throughout the biome and function as areas of water accumulation and drainage for rivers and watersheds, playing a crucial role in maintaining regional hydrological systems (Horák-Terra et al. 2022; Durigan et al. 2022).

*Vereda* ecosystems harbor unique biodiversity and exhibit a complex open vegetation structure, composed of a rich assemblage of species that reflects the strong ecological filtering imposed by flooding and fire regimes (Araújo et al. 2002; Ribeiro and Walter 2008; Oliveira et al. 2009; Ávila et al. 2016; Sabino et al. 2021). Consequently, both vegetation composition and the phenological patterns of these communities are closely associated with hydrological dynamics characteristic of these environments (Luna et al. 2024). However, like other Cerrado formations, *Veredas* have experienced human disturbance and hydrological alterations over recent decades as a result of intensive water use and land-use changes in adjacent areas (Hofmann et al. 2021; Nunes et al. 2022; Gonçalves et al. 2022 ; dos Santos et al. 2025a, b).

As a consequence, *Vereda* vegetation, which is primarily composed of a herbaceous-grass layer with or without the presence of the buriti palm (*Mauritia flexuosa*), and is structurally organized and spatially zoned according to soil moisture gradients, has undergone structural and functional changes commonly referred to as *Vereda* drought (Nunes et al. 2022). Despite the growing attention devoted to this phenomenon, its underlying mechanisms and ecological consequences remain poorly understood (Passos et al. 2025; Horák-Terra et al. 2022; Bijos et al. 2023).

Among the main changes associated with *Vereda* drought is the establishment and expansion of woody species originating from the drier surrounding Cerrado vegetation (Horák-Terra et al. 2022; Nunes et al. 2022; Trindade et al. 2024). This process, known as woody plant encroachment (WPE), has been widely documented across non-forest tropical ecosystems (Deng et al. 2021; Gonçalves et al. 2021). In *Veredas*, WPE has altered vegetation structure and species composition, promoting the exclusion of certain taxa and reducing the functional diversity of plant communities, thereby threatening the ecological integrity of these ecosystems (Silva et al. 2016; Trindade et al. 2024).

These changes associated with WPE may affect the entire network of ecological interactions within *Veredas*, including pollinators, which depend on both the availability and temporal synchronization of floral resources provided by plants (Luna et al. 2024). Because the supply of these resources is regulated by species phenology, alterations in vegetation composition and structure may modify the temporal patterns of resource availability throughout the year (Luna et al. 2024; Santos et al. 2025a, b). Furthermore, shifts in phenological patterns may influence the seasonal dynamics of photosynthetic activity and, consequently, the primary productivity of *Vereda* ecosystems, with potential implications for ecosystem functioning and the maintenance of biotic interactions in these environments.

Despite the importance of phenology for maintaining ecological processes in *Veredas*, little is known about how WPE affects the temporal dynamics of native vegetation in these ecosystems. Therefore, understanding the effects of this process on phenological patterns and primary productivity is essential. In this study, we integrated high-resolution imagery acquired by a Remotely Piloted Aircraft System (RPAS) with field monitoring to characterize the seasonal patterns of flowering and productivity over one year in species associated with WPE and in native *Vereda* vegetation. Specifically, we addressed the following questions: (1) Do species associated with woody plant encroachment exhibit phenological and primary productivity patterns distinct from those observed in native *Vereda* vegetation? (2) Does the presence of these species alter the temporal dynamics of flowering in native *Vereda* vegetation? We hypothesized that species associated with woody plant encroachment display vegetative and reproductive phenological patterns that differ from those of native *Vereda* vegetation. Consequently, their expansion modifies the seasonality of floral resource availability and primary productivity patterns, promoting a temporal homogenization of community phenological dynamics.

## Materials and methods

### Study area

**Fig. 1:**
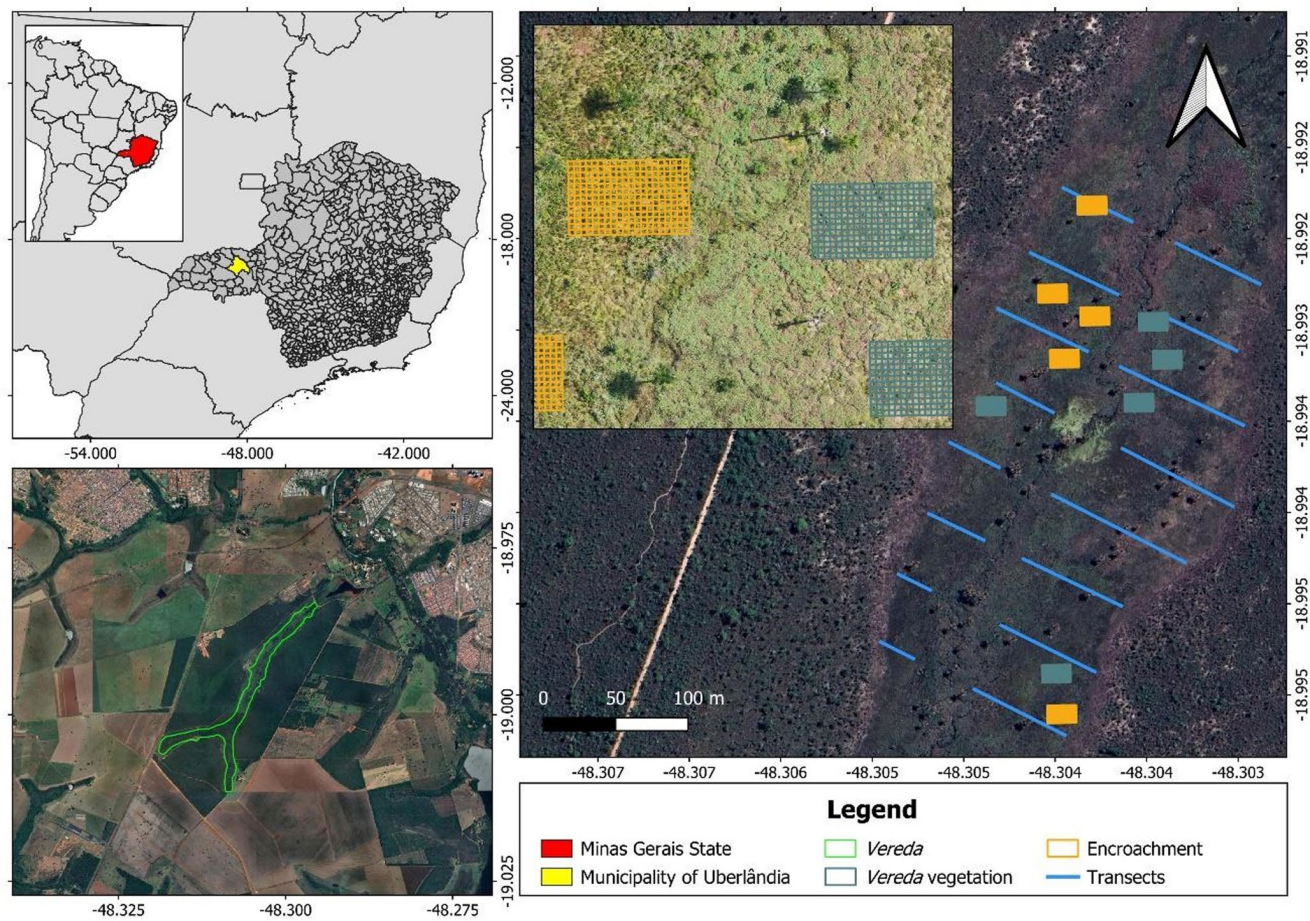
Sampling area in the *Vereda* of the Itororó hunting and fishing club of Uberlândia (CCPIU) reserve, located in Uberlândia, Minas Gerais, Brazil (18°59′35.11″ S, 48°18′13.72″ W). The grid rectangles represent the plots, native *Vereda* vegetation is shown in blue, WPE areas in orange, and flowering phenology transects are represented by blue lines

This study was conducted in a *Vereda* of the private conservation area of the Itororó Hunting and Fishing Club of Uberlândia (CCPIU), Uberlândia, Minas Gerais state, in southeastern Brazil (18°59′35.11″ S, 48°18′13.72″ W). The reserve has approximately 400 hectares, with a predominance of Cerrado stricto sensu (Ribeiro and Walter 2008) and a 74-hectare *Vereda* (Araújo et al. 2002). The regional climate is strongly seasonal, with a rainy season typically extending from October to March and a dry season from April to September, during which precipitation is substantially reduced (Araújo et al. 2002; Sabino et al. 2021). These seasonal rainfall fluctuations represent the main climatic driver of vegetation dynamics in Cerrado ecosystems (Ribeiro and Walter 2008). The *Vereda* crosses the entire area, occupying a shallow valley with a continuous herbaceous layer, interspersed by shrubs, small trees (Fig. 1), and the iconic buriti palm (*Mauritia flexuosa*). The *Veredas* can be subdivided into roughly parallel zones in relation to the main watercourse: a drier edge zone, an intermediate middle zone, and a frequently flooded center (Araújo et al. 2002). Along the last two decades, despite being protected, the area has been affected by frequent frosts, fires, and deforestation of its surroundings for agricultural use (Barbosa 2021).

### RPAS-based phenological data collection

To obtain vegetative phenological data from woody plant encroachment (WPE) areas and native *Vereda* vegetation, monthly RPAS surveys were conducted over twelve consecutive months, from September 2022 to August 2023, using a DJI Mavic 2 Pro drone. Flights were performed during the last week of each month, preferentially on days with clear-sky conditions and stable illumination, at approximately 10:00 a.m., in order to minimize variation in solar angle and atmospheric conditions among sampling dates. Flights were conducted at an altitude of 100 m, with 75% front overlap and 65% side overlap, at a flight speed of 8 m s⁻¹, and the camera positioned at − 90° relative to the ground. Each flight campaign generated approximately 480 RGB images, which were processed using Pix4D Mapper software version 4.4.12 (PIX4D 2020). The resulting orthomosaics had a spatial resolution of 5 cm per pixel and were exported in GeoTIFF (.tif) format using the WGS 84/UTM Zone 22 S coordinate reference system (EPSG:32722), maintaining the same spatial reference across all acquisition dates. The RPAS surveys covered approximately 731,175 m² (73.1 ha) per flight, encompassing the entire study landscape, including the *Vereda* and adjacent Cerrado vegetation. Within this area, the mapped *Vereda* occupied approximately 178,972 m² (17.9 ha), which served as the spatial reference for subsequent phenological analyses.

### Identification of woody plant encroachment species and flowering phenology collection

**Fig. 2:**
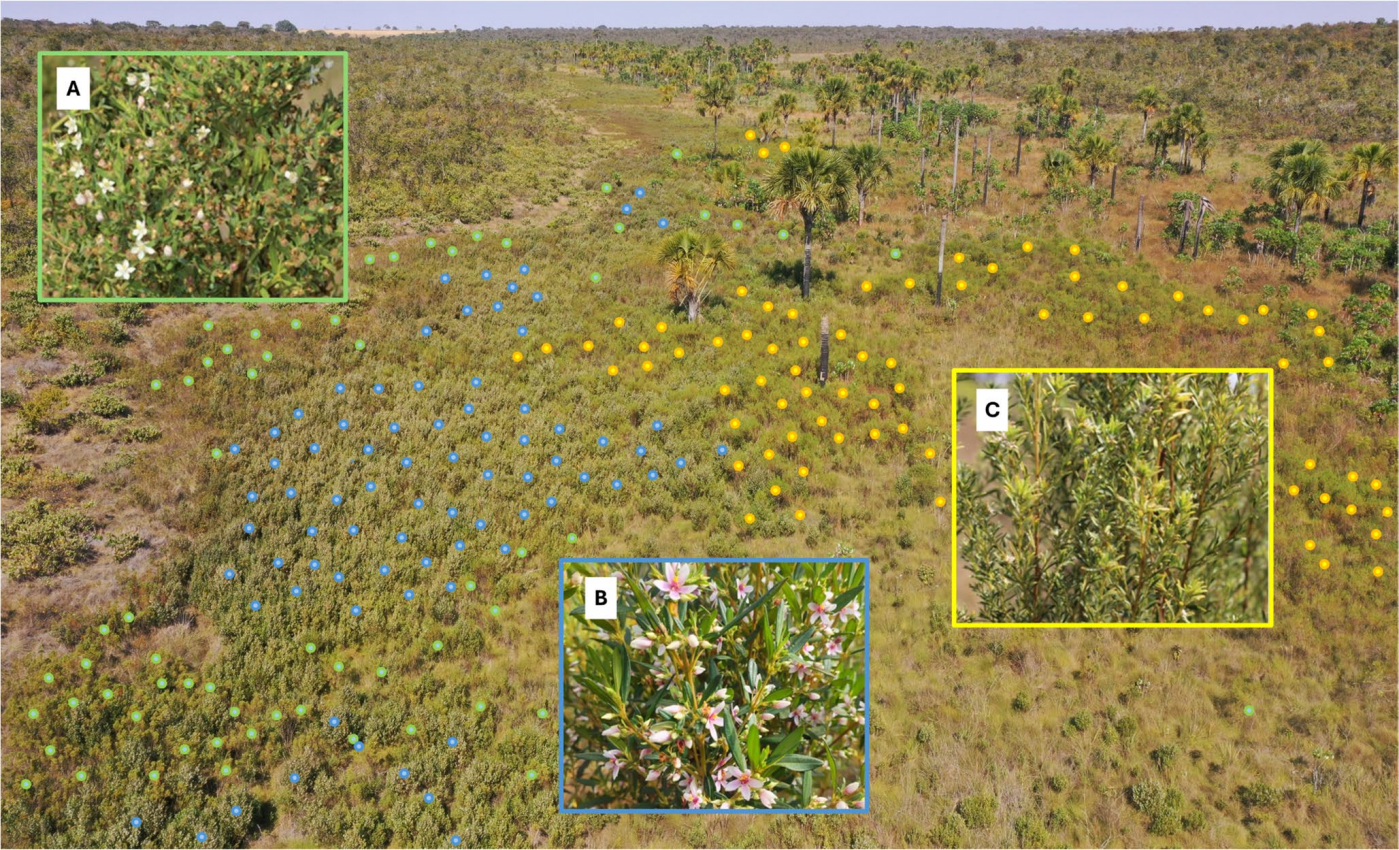
Species of woody plant encroachment in the *Vereda* reserve of the Itororó hunting and fishing club of Uberlândia (CCPIU). (**A**) *Microlicia phlogiformis* (Melastomataceae), (**B**) *Microlicia parviflora* (Melastomataceae) and (**C**) *Baccharis dracunculifolia* (Asteraceae)

Shrub species associated with woody plant encroachment (WPE) were identified through field surveys conducted in 2023, during which georeferenced points corresponding to the centroids of WPE patches were recorded to facilitate the spatial identification of WPE areas in the RPAS imagery. Botanical material was collected for taxonomic identification and voucher specimen preparation. Voucher specimens were deposited in the Herbarium Uberlandense (HUFU) at the Institute of Biology, Federal University of Uberlândia. The species responsible for the WPE were *Baccharis dracunculifolia* (Asteraceae), *Microlicia phlogiformis* (Melastomataceae), and *Microlicia parviflora* (Melastomataceae) (Fig. 2). These WPE species have been recorded in other *Vereda* areas in Central Brazil (e.g., Costa et al. 2023) and form largely monodominant patches, which cover some 13% of the middle portion of the studied *Vereda* (J.P. Costa and N. Alves unpublished data). These WPE patches have been increasing along the last decade and tend to cover and exclude native plants (Fig. 2).

Flowering phenology was monitored from January to December 2023 along 5 m wide transects arranged transversely across the middle zone of the Vereda, with bi-weekly visual observations. Transect lengths varied from 152.5 to 203.1 m, resulting in a total sampled area of approximately 7,102.2 m². For each fortnight, we recorded the number of individuals exhibiting flowers for each species within the entire sampled area. Only flowering individuals were considered in the phenological analyses; non-flowering individuals were not counted. The monitored flora included three WPE species (Baccharis dracunculifolia, Microlicia parviflora, and Microlicia phlogiformis) and the remaining species of native vegetation occurring in the middle zone of the Vereda, totaling 140 flowering species recorded during the study period. As WPE species occurred exclusively in this zone, all comparative analyses were restricted to this microhabitat. The flowering individuals of all native species were pooled, excluding the WPE species. For each group, data were aggregated by fortnight by summing all flowering individuals across the sampled area, regardless of transect, resulting in time series describing flowering activity for WPE species and for the remaining native Vereda species.

### Definition of regions of interest and spatial analyses design

Ten regions of interest (ROIs) were defined to characterize vegetation phenological patterns. They were marked in areas previously defined in the field as areas of woody plant encroachment (WPE), but we sought to locate five ROis in areas of WPE and five ROIs in native *Vereda* vegetation, as close as possible to the WPE ROIs. Each ROI covered an area of 260 m², within which a regular grid of 1 × 1 m plots was generated (Fig. 1). Thus, each ROI comprised approximately 260 plots. The sampling grids were generated using QGIS (2024) software (version 3.34) and stored as vector layers in GeoPackage (.gpkg) format, ensuring precise spatial correspondence between all plots and the orthomosaics throughout the temporal series. Each plot was considered an independent sampling unit for the extraction of spectral indices.

### Extraction of the green chromatic coordinate (GCC)

The Green Chromatic Coordinate (GCC) was used as an indicator of vegetation phenological dynamics. This index allows the detection of variations in green reflectance relative to the red and blue primary colors and has been widely applied to infer fluctuations in leaf productivity and vegetation phenology (Alberton et al. 2014; Deng et al. 2021). Because GCC is closely associated with changes in canopy greenness, leaf development, chlorophyll content, and photosynthetically active vegetation, increases in GCC generally indicate periods of leaf flushing and greater vegetative activity, whereas decreases are commonly associated with leaf senescence and reduced canopy productivity. GCC was calculated from the RGB bands of the orthomosaics according to the following equation:

$$\:{G}_{cc}=\frac{Green}{Red+Green+Blue}$$

where R, G, and B corresponded to the red, green, and blue bands, respectively.

GCC values were extracted in the Google Colab environment using the Python programming language and the “*rasterio*”, “*geopandas*”, “*numpy*”, and “*pandas*” libraries. For each month and each 1 m² plot, raster values were clipped according to plot geometry, and the mean GCC value was calculated. This procedure resulted in a dataset representing the monitored plots over 12 months, encompassing both woody plant encroachment (WPE) areas and native *Vereda* vegetation.

### Statistical analysis

To test for differences in greenness magnitude and seasonal phenological trajectories between woody plant encroachment (WPE) and native *Vereda* areas, we fitted linear mixed-effects models (LMMs). The Green Chromatic Coordinate (GCC) was used as the response variable. Vegetation type (*Vereda* and WPE), time (months), and their interaction were included as fixed effects to assess both overall differences in GCC and temporal variation in phenological patterns. Plot identity (1 × 1 m sampling units) was included as a random intercept to account for repeated measurements through time. Models were fitted using restricted maximum likelihood (REML) implemented in the “*statsmodels”* package in Python. Statistical significance of fixed effects was assessed using z-tests, with a significance threshold of p = 0.05.

The model was specified as:

$$\begin{aligned}&\\&\:{GCC}_{ij}={\beta\:}_{0}+{\beta\:}_{1}{Vegetation\:type}_{i}+{\beta\:}_{2}{Time}_{j}&\\&+{\beta\:}_{3}({Vegetation\:type}_{i}\times\:{Time}_{j})+{u}_{i}+{\epsilon\:}_{ij}\end{aligned}$$

where $\:{u}_{i}$ represented the random effect associated with plot $\:i$ and $\:{\epsilon\:}_{ij}$the residual error.

For flowering phenology, due to the cyclical nature of the data, analyses were conducted using circular statistical methods (Morellato et al. 2010; Hart et al. 2025). Each fortnight was converted into an angle, such that the first fortnight corresponded to 0 rad and the 24th fortnight to 2π rad. For each species or group, the mean angle, representing the central flowering period, and the concentration parameter *r*, ranging from 0 to 1 and indicating the degree of flowering synchrony throughout the year, were calculated. To test whether the flowering phenology of WPE species differed from that of native *Vereda* species, a permutation test was implemented in Python using the *“pandas”* package for data manipulation, *“numpy”* for numerical computations, and *“matplotlib”* for visualization. The test consisted of calculating the observed difference in mean flowering angle between the groups. Individuals were then randomly reassigned between groups across 10,000 permutations, recalculating the mean difference at each iteration. The empirical *p*-value was obtained as the proportion of permutations in which the simulated difference was equal to or greater than the observed difference, allowing inference on whether flowering patterns differed significantly between the groups (Good 2005).

Because GCC values were extracted from contiguous 1 × 1 m plots, additional analyses were performed to evaluate potential spatial dependence among neighboring observations. The use of fine-scale plots was motivated by the spatial structure of WPE, which occurs as dense vegetation patches composed of closely spaced individuals, often resulting in multiple individuals occurring within a single square meter. Consequently, some degree of spatial autocorrelation was expected and reflects the biological organization of the vegetation rather than a sampling artifact.

Spatial autocorrelation was quantified using Moran’s I, calculated separately for each study area and monthly orthomosaic. Analyses were based on plot centroid coordinates and a distance-based neighborhood matrix connecting plots within a 1.5 m threshold. Statistical significance was assessed using 999 random permutations. To evaluate whether differences between vegetation types could be influenced by spatial pseudoreplication, a complementary analysis was conducted using the ten regions of interest (ROIs) as independent replicates. Mean GCC values were calculated for each ROI, and annual phenological metrics, including mean GCC, maximum GCC, minimum GCC, phenological amplitude, and temporal coefficient of variation, were derived from the monthly time series. Differences between *Vereda* and WPE areas were subsequently evaluated using Welch’s t-tests, Mann–Whitney U tests, and Cohen’s d effect sizes.

## Results

### Vegetation greenness dynamics and spatial structure

The linear mixed-effects model revealed significant differences in GCC values between vegetation types. Woody plant encroachment (WPE) areas exhibited significantly higher GCC values than native *Vereda* areas (β = 0.013 ± 0.001 SE; z = 25.47; *p* < 0.001). In addition, a significant interaction between vegetation type and time was detected (β = 0.002 ± 0.000 SE; z = 24.12; *p* < 0.001), indicating that temporal trajectories of vegetation greenness differed between WPE and *Vereda* areas.

**Fig. 3:**
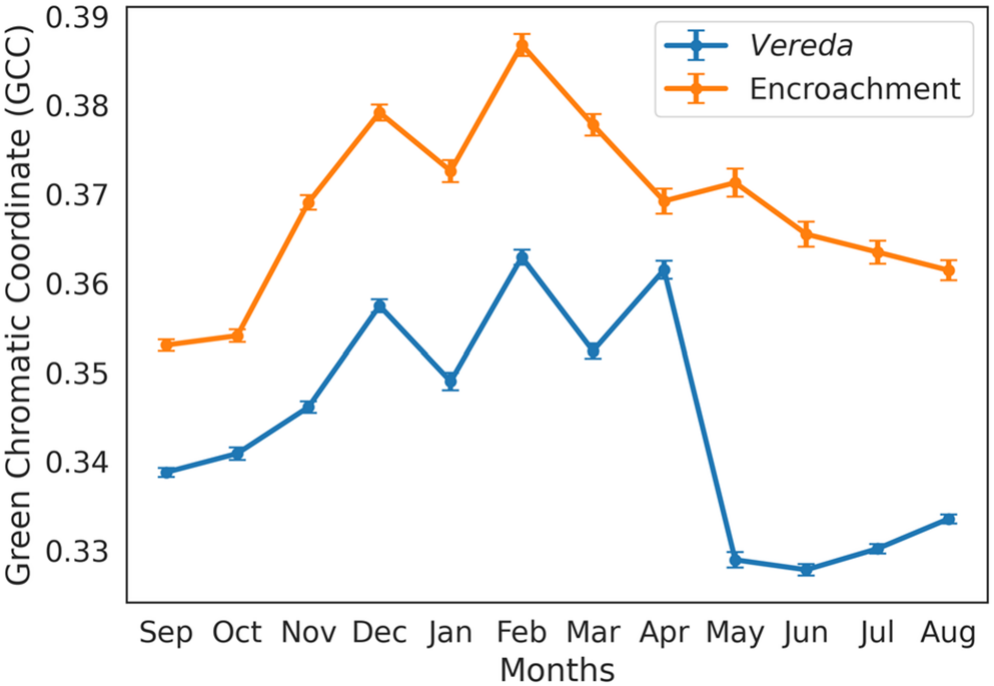
Seasonal dynamics of the green color coordinate (GCC) in *Vereda* and woody plant encroachment (WPE) areas from September/2022 to August/2023. The points represent the monthly averages of GCC calculated from 1 m² plots, and the vertical bars indicate the 95% confidence intervals

Monthly GCC values showed that WPE areas maintained consistently higher greenness throughout the annual cycle, particularly during transitions between the dry season (April–September) and the rainy season (October–March), which characterize the strongly seasonal climate of the Cerrado. Although WPE areas consistently exhibited higher GCC values, temporal trajectories differed between vegetation types. During the transition from the rainy season to the dry season (March–April), GCC values increased slightly in native *Vereda* vegetation while declining in WPE areas, temporarily reducing the difference between environments. Subsequently, native *Vereda* vegetation exhibited a sharper decline in GCC during the dry season, whereas WPE areas maintained comparatively higher greenness levels. WPE areas also displayed higher average GCC values and steeper increases in greenness during recovery periods, indicating altered vegetation dynamics relative to native *Vereda* communities. Variance associated with the random effect of plot was close to zero, indicating high temporal consistency of GCC measurements within plots and a strong signal-to-noise ratio in the RPAS-derived spectral data (Fig. 3).

Because GCC was extracted from contiguous 1 × 1 m plots, we further evaluated whether spatial dependence among neighboring plots could influence these results. Moran’s I analyses revealed significant positive spatial autocorrelation across all study areas and sampling periods. Mean Moran’s I values ranged from 0.379 to 0.515 among areas, with individual monthly values varying between 0.136 and 0.697 (Table S1). These results indicate moderate to strong spatial structuring of GCC, with neighboring plots tending to exhibit similar greenness values. Such spatial dependence was expected given the ecological organization of WPE, which occurs as dense patches of closely spaced woody shrubs occupying contiguous portions of the landscape.

To determine whether the observed differences between environments were driven by spatial pseudoreplication, GCC values were subsequently aggregated at the area level, reducing the dataset from 2,600 plots to 10 independent study areas (five *Veredas* and five WPE areas). The difference between vegetation types remained significant when study areas were treated as the units of replication. Mean annual GCC was lower in *Veredas* (0.346 ± 0.006) than in WPE areas (0.367 ± 0.008) (Welch’s t-test: t = −4.786, *p* = 0.0017; Mann–Whitney U test: U = 0, *p* = 0.0079). The effect size was extremely large (Cohen’s d = 3.03). Notably, all WPE areas exhibited higher annual GCC values than all *Vereda* areas, demonstrating complete separation between environments at the area scale. These results indicate that the higher greenness observed in WPE areas cannot be attributed solely to spatial dependence among neighboring plots, but instead reflects a consistent ecological difference between vegetation types.

Phenological metrics derived from area-level GCC further revealed that WPE areas maintained higher greenness throughout the annual cycle. WPE areas exhibited higher annual mean GCC, higher maximum GCC, and higher minimum GCC values than *Veredas*. Annual mean GCC differed significantly between environments (*p* = 0.0017; Cohen’s d = 3.03), as did maximum GCC (*p* = 0.0055; Cohen’s d = 2.49) and minimum GCC (*p* = 0.0046; Cohen’s d = 3.43) (Table S3). In contrast, temporal variability metrics showed substantial overlap between environments. No significant differences were detected for phenological amplitude (*p* = 0.9823; Cohen’s d = −0.01) or temporal coefficient of variation (*p* = 0.4029; Cohen’s d = −0.57) (Table S3). Phenological amplitude, calculated as the difference between annual maximum and minimum GCC values, ranged from 0.021 to 0.040 in *Veredas* and from 0.022 to 0.045 in WPE areas. Similarly, temporal coefficients of variation ranged from 2.0 to 4.1% in *Veredas* and 1.9–3.6% in WPE areas (Table S2).

### Flowering phenology

**Fig. 4:**
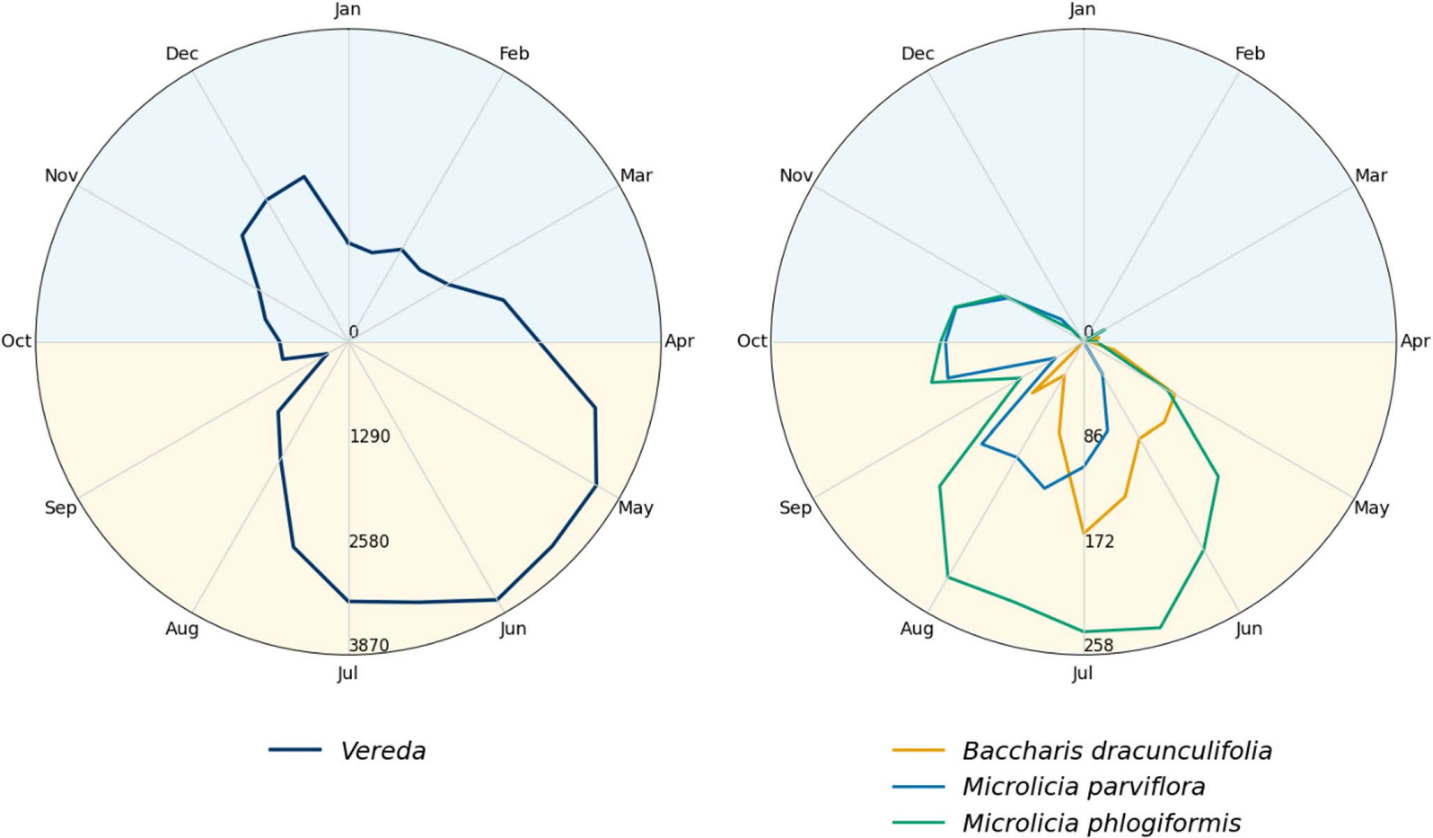
Flowering phenological patterns in the *Vereda* middle area/zone for woody plant encroachment (WPE) and native *Vereda* species. The left panel represents the overall flowering of the *Vereda*, considering only species occurring in the middle zone and WPE species. The right panel shows the flowering patterns of the three WPE species (*Baccharis dracunculifolia*, *Microlicia parviflora*, and *Microlicia phlogiformis*). Circular plots represent the summed number of flowering individuals across 24 fortnightly periods distributed throughout the year, with months indicated radially. Shaded areas indicate the rainy season (blue; October to March) and the dry season (yellow; April to September). Polygons depict temporal variation in flowering

Flowering phenology revealed a distinct pattern from that observed for GCC. Species associated with woody plant encroachment (WPE) exhibited a highly concentrated flowering period throughout the annual cycle. When analyzed jointly, WPE species presented a mean flowering angle of 3.45 rad and a high resultant vector length (*r* = 0.65), indicating strong temporal synchrony and well-defined flowering peaks. In contrast, native *Vereda* species occurring in the middle zone displayed a much broader temporal distribution of flowering events. The *Vereda* community showed a mean flowering angle of 1.99 rad and a substantially lower resultant vector length (*r* = 0.27), reflecting high interspecific asynchrony and the absence of a dominant flowering peak at the community level (Fig. 4).

The difference between the mean flowering angles of WPE and *Vereda* species corresponded to a temporal displacement of approximately three months. However, this difference was not statistically significant according to the circular permutation test (*p* = 0.229). Despite the lack of statistical significance, the markedly higher vector length observed in WPE species indicates a substantially more synchronized reproductive phenology relative to the native *Vereda* flora.

## Discussion

Our results indicate that areas affected by woody plant encroachment (WPE) exhibit phenological patterns that differ substantially from those of native *Vereda* vegetation, encompassing both vegetative and reproductive dimensions. WPE areas maintained consistently higher vegetation greenness throughout the annual cycle, exhibiting higher mean, maximum, and minimum GCC values than native *Veredas*. However, despite these differences in greenness magnitude, no significant differences were detected in phenological amplitude or temporal variability, indicating that WPE increases vegetation greenness throughout the year without substantially altering the magnitude of seasonal fluctuations. In parallel, species associated with WPE exhibited markedly greater flowering synchrony than the native *Vereda* community, revealing changes not only in vegetation activity but also in the temporal organization of reproductive events. Together, these findings suggest that WPE does not eliminate the seasonal phenological dynamics characteristic of *Veredas*, but shifts the entire system toward a state characterized by higher year-round greenness, greater dominance of a few woody species, and increased reproductive synchrony.

The robustness of these patterns was further supported by additional spatial analyses. Although significant spatial autocorrelation was detected among neighboring plots, as expected for vegetation organized in dense and spatially continuous patches, the differences between WPE and native *Vereda* areas remained significant when analyses were repeated using the ten study areas as independent replicates. The persistence of these differences, coupled with very large effect sizes, indicates that the observed phenological shifts are not artifacts of spatial dependence among neighboring plots but represent consistent ecological differences between vegetation states. Importantly, the maintenance of these patterns after aggregation at the ROI level suggests that the detected phenological changes emerge at the scale of vegetation patches and landscape organization rather than merely reflecting fine-scale spatial variation among adjacent sampling units.

*Veredas* are among the most distinctive wetland ecosystems of the Cerrado, characterized by permanent or seasonal water saturation, strong hydrological control of ecological processes, and the occurrence of highly specialized plant communities adapted to fluctuating water availability (Honda and Durigan 2016; Sabino et al. 2021). In these ecosystems, seasonal hydrological pulses associated with the alternation between the rainy season (October–March) and the dry season (April–September) act as major drivers of vegetation activity and reproductive timing. Against this ecological background, the phenological shifts observed in WPE areas likely represent more than simple changes in vegetation composition; they may indicate an ongoing reorganization of ecosystem functioning. The replacement of diverse native assemblages by dense woody stands modifies not only the structural attributes of the vegetation but also the temporal dynamics through which ecological processes unfold.

The increase in woody biomass tends to intensify water demand throughout the year, especially during the dry season, when *Veredas* play a fundamental role in maintaining soil moisture and regulating the discharge of associated watercourses (Honda and Durigan 2016). Such changes may reduce surface and subsurface water availability, compromising key ecosystem functions of this wetland environment and potentially favoring further WPE (Ribeiro et al. 2020; Nunes et al. 2022). The GCC patterns observed in this study support this interpretation. Rather than exhibiting a reduction in seasonality, WPE areas maintained higher vegetation greenness during both favorable and unfavorable periods of the year. The significantly higher minimum GCC values observed in WPE areas suggest that woody species remain physiologically active for longer periods, likely due to greater access to deep soil water reserves and longer leaf lifespan (Oliveira et al. 2021; Deng et al. 2021). This pattern may contribute to sustained transpiration and water consumption throughout the year, reinforcing drying processes and altering hydrological feedbacks that are fundamental to *Vereda* persistence (Schreiner-McGraw et al. 2020; Nunes et al. 2022).

This distinct phenological behavior likely reflects the functional replacement of herbaceous and graminoid species, which typically exhibit strong seasonal reductions in leaf area and photosynthetic activity, by woody shrubs capable of maintaining foliage and physiological activity for longer periods. Although GCC amplitude remained similar between environments, WPE areas consistently operated at a higher baseline of vegetation greenness. Consequently, WPE appears to shift the overall level of vegetation activity rather than fundamentally altering its seasonal rhythm. This distinction is ecologically important because it suggests that the strong seasonal rainfall regime characteristic of the Cerrado continues to influence vegetation dynamics, but the response of the plant community occurs around a new functional baseline. Such a shift may represent an early stage of ecosystem transformation, preceding more pronounced changes in hydrology, nutrient cycling, and vegetation structure.

Changes in vegetative dynamics were accompanied by marked alterations in reproductive phenology. Species associated with WPE exhibited substantially greater flowering synchrony than native *Vereda* species, indicating a concentration of reproductive activity within narrower temporal windows. Although the difference in mean flowering date between groups was not statistically significant, the much higher concentration parameter observed in WPE species demonstrates that flowering events became considerably more synchronized under encroachment. This result is particularly noteworthy because flowering synchrony may influence pollinator behavior, pollen transfer efficiency, reproductive success, and the temporal availability of floral resources (Luna et al. 2024; Santos et al. 2025a, b; Terasaki et al. 2025).

The higher flowering synchrony observed in WPE areas should also be interpreted in the context of vegetation homogenization associated with encroachment. While flowering analyses for WPE areas included only three species, these species represent the dominant woody components responsible for the encroachment process and occupy extensive portions of the landscape. Thus, the reduced number of species is itself a consequence of encroachment rather than a methodological limitation. In contrast, native *Vereda* communities comprise a more diverse assemblage of species with distinct flowering schedules, resulting in lower community-level synchrony and a more continuous distribution of floral resources throughout the year. The dominance of a few abundant woody species therefore promotes not only structural homogenization of the vegetation but also homogenization of reproductive dynamics and floral resource availability.

This process can be viewed within the broader framework of biotic and functional homogenization. WPE simplifies community composition by increasing the dominance of a small number of species while reducing the contribution of diverse native taxa (Honda and Durigan 2016). As a consequence, temporal variation in ecological functions may also become simplified. In native *Veredas*, the asynchronous flowering of multiple species generates a temporally distributed supply of floral resources, potentially supporting a wider diversity of pollinators and other interacting organisms throughout the year (Cardoso et al. 2026). In contrast, the greater synchrony observed in WPE areas suggests a concentration of resource availability within shorter periods, potentially reducing temporal niche complementarity and increasing dependence on a narrower set of phenological windows.

Alterations in flowering phenology may therefore generate cascading effects on ecological interactions. Increased synchrony and shifts in flowering timing can modify the availability, duration, and temporal continuity of floral resources, potentially affecting pollinator assemblages and disrupting the coupling between plants and their mutualists (Luna et al. 2024; Santos et al. 2025a, b; Terasaki et al. 2025). In ecosystems such as *Veredas*, where seasonal fluctuations in rainfall and hydrology act as principal organizing forces of ecological interactions, these changes may have disproportionate effects on interaction networks and functional diversity. Together with the persistently elevated greenness observed in WPE areas, the concentration of flowering periods suggests an ongoing process of functional and phenological homogenization. Although seasonal GCC amplitude remained similar between environments, the combination of higher year-round greenness and more synchronized flowering may reduce temporal heterogeneity in resource availability and compromise ecosystem resilience.

From an ecophysiological perspective, the consistently higher mean, maximum, and minimum GCC values observed in WPE areas indicate the maintenance of elevated vegetation activity throughout the year and, consequently, a more continuous demand for resources, particularly water. In environments naturally regulated by seasonal hydrological pulses associated with alternating wet and dry periods, such as *Veredas*, this shift may intensify evapotranspiration during the dry season and reduce the duration or intensity of surface waterlogging periods (Honda and Durigan 2016; Acharya et al. 2018). Such changes may create positive feedbacks that further favor woody species capable of maintaining physiological activity during water-limited periods, reinforcing the encroachment process and accelerating ecosystem transformation (Silva et al. 2016). In this sense, phenological shifts may not merely be indicators of encroachment but active components of the mechanisms through which encroachment progresses and stabilizes over time.

Finally, our results reinforce the potential of RPAS-derived GCC as a sensitive indicator of fine-scale phenological changes in Cerrado wetland ecosystems. The persistence of GCC differences after accounting for spatial autocorrelation demonstrates that RPAS-based phenological metrics are capable of detecting biologically meaningful shifts associated with WPE. Interestingly, GCC data showed particularly clear contrasts between WPE and native *Vereda* areas during the dry season, whereas multispectral approaches have been reported to perform better during wetter periods in other *Vereda* systems (Costa et al. 2023). The integration of high-resolution spectral metrics with field-based phenological observations, including flowering dynamics, provides a powerful framework for detecting early functional changes in *Veredas* and for supporting monitoring, management, and restoration strategies aimed at maintaining their ecological integrity and natural phenological regimes.

Some limitations of this study should be acknowledged. Phenological observations were conducted over a single annual cycle, and interannual climatic variability may influence both vegetative and reproductive phenological patterns. In addition, the study was restricted to a single *Vereda*, although the monitored area encompassed a substantial portion of the wetland and included both preserved and woody-encroached sectors. Consequently, caution should be exercised when extrapolating these findings to other *Veredas* across the Cerrado biome. Despite these limitations, the consistency between RPAS-derived GCC patterns and field observations highlights the robustness of the approach and its potential for broader application. Future studies extending monitoring across multiple years and multiple *Veredas*, while integrating phenology, hydrology, and plant–pollinator interactions, may help clarify whether the observed increases in greenness and flowering synchrony ultimately translate into long-term changes in ecosystem resilience, biodiversity maintenance, and wetland functioning.

## Conclusion

This study provides evidence that woody plant encroachment (WPE) can alter the phenological dynamics of *Vereda* vegetation, affecting both vegetative and reproductive processes. In the studied *Vereda*, WPE areas maintained consistently higher greenness and reduced seasonal variation compared to native vegetation, indicating shifts in vegetation functioning associated with the increasing dominance of woody species. WPE was also associated with more synchronized and temporally concentrated flowering patterns than those observed in the native *Vereda* community, suggesting a tendency toward phenological homogenization within encroached areas.

Although these findings are restricted to a single *Vereda* and one annual monitoring cycle, they indicate that WPE has the potential to modify multiple dimensions of ecosystem phenology, with possible consequences for resource dynamics and ecological interactions. The integration of high-resolution RPAS imagery with field-based phenological observations proved effective for detecting these fine-scale changes and represents a promising approach for monitoring vegetation transitions in Cerrado wetlands. Future studies encompassing multiple *Veredas*, longer temporal series, and different encroaching species will be important to evaluate the generality of these patterns across the Cerrado biome.

## Supplementary Information

Below is the link to the electronic supplementary material.

Supplementary Material 1 (CSV 7.07 KB)

Supplementary Material 2 (CSV 1.17 KB)

Supplementary Material 3 (CSV 566 bytes)

## References

[CR1] Acharya BS, Kharel G, Zou CB, Wilcox BP, Halihan T (2018) Woody plant encroachment impacts on groundwater recharge: A review. Water 10:1466. 10.3390/w10101466

[CR2] Alberton B, Almeida J, Helm R, Torres RS, Menzel A, Morellato LPC (2014) Using phenological cameras to track the green up in a cerrado savanna and its on-the-ground validation. Ecol Inform 19:62–70. 10.1016/j.ecoinf.2013.12.011

[CR4] Araújo GM, Barbosa AA, Arantes AA, Amaral AF (2002) Composição florística de veredas no Município de Uberlândia, MG. Braz J Bot 25:475–493. 10.1590/S0100-84042002000400012

[CR5] Ávila MA, Lazarotto M, Brião Muniz MF, Girardi LB, Lippert DB, Maciel CG (2016) Structure of natural regeneration in relation to soil properties and disturbance in two swamp forests. Cerne 22:1–10. 10.1590/01047760201622012086

[CR6] Barbosa L (2021) Incêndio destrói quase 600 hectares de reserva no Clube Caça e Pesca. Diário de Uberlândia. https://diariodeuberlandia.com.br/noticia/29364/incendio-destroi-quase-600-hectares-de-reserva-no-clube-caca-e-pesca. Accessed 17 Jan 2026

[CR7] Bijos NR, Silva DP, Munhoz CBR (2023) Soil texture and fertility determine the beta diversity of plant species in veredas in Central Brazil. Plant Soil 492:241–259. 10.1007/s11104-023-06168-3

[CR9] Cardoso JCF, Trevizan R, Maruyama PK, Caetano APS, Gonçalves RVS, Antonini Y, Oliveira PE (2026) Pollination and plant reproduction in the Cerrado, the world’s most biodiverse savanna. Biol Rev 101:74–105. 10.1111/brv.70073 40957635 10.1111/brv.70073 PMC12783448

[CR10] Costa LS, Sano EE, Ferreira ME, Munhoz CBR, Costa JVS, Rufino Alves Júnior L et al (2023) Woody plant encroachment in a seasonal tropical savanna: lessons about classifiers and accuracy from UAV images. Remote Sens 15:2342. 10.3390/rs15092342

[CR11] Deng Y, Li X, Shi F, Hu X (2021) Woody plant encroachment enhanced global vegetation greening and ecosystem water-use efficiency. Glob Ecol Biogeogr 30:2337–2353. 10.1111/geb.13386

[CR13] Durigan G, Munhoz CB, Zakia MJB, Oliveira RS, Pilon NA, do Valle RST et al (2022) Cerrado wetlands: multiple ecosystems deserving legal protection as a unique and irreplaceable treasure. Perspect Ecol Conserv 20:185–196. 10.1016/j.pecon.2022.06.002

[CR14] Gonçalves RVS, Cardoso JCF, Oliveira PE, Oliveira DC (2021) Changes in the Cerrado vegetation structure: insights from more than three decades of ecological succession. Web Ecol 21:55–64. 10.5194/we-21-55-2021

[CR15] Gonçalves RVS, Cardoso JCF, Oliveira PE, Raymundo D, De Oliveira DC (2022) The role of topography, climate, soil and the surrounding matrix in the distribution of Veredas wetlands in central Brazil. Wetl Ecol Manag 30:1261–1279. 10.1007/s11273-022-09890-9

[CR16] Good PI (2005) Permutation, parametric, and bootstrap tests of hypotheses. Springer, New York

[CR37] Hart DET, Bùi T-N, Di Maggio L, Wang IJ (2025). Global phenology maps reveal the drivers and effects of seasonal asynchrony. Nature, 645, 133–140. 10.1038/s41586-025-09410-3 10.1038/s41586-025-09410-3 PMC12408380 40866701

[CR17] Hofmann GS, Cardoso MF, Alves CRJV et al (2021) The Brazilian Cerrado is becoming hotter and drier. Glob Change Biol 27:4060–4073. 10.1111/gcb.15712 10.1111/gcb.15712 34018296

[CR18] Honda EA, Durigan G (2016) Woody encroachment and its consequences on hydrological processes in the savannah. Philos Trans R Soc B Biol Sci 371:20150313. 10.1098/rstb.2015.0313 10.1098/rstb.2015.0313 PMC4978871 27502378

[CR19] Horák-Terra I, Terra FS, Lopes AKA et al (2022) Soil characterization and drainage effects in a savanna palm swamp (vereda) of an agricultural area from Central Brazil. Rev Bras Cienc Solo 46:e0210065. 10.36783/18069657rbcs20210065

[CR20] Luna ALL, Souza CS, Neves JGS et al (2024) Temporal and spatial variation of floral resources of woody species in a vereda ecosystem: uniformity and habitat complementarity. Flora 310:152425. 10.1016/j.flora.2023.152425

[CR21] Morellato LPC, Alberti LF, Hudson IL (2010) Applications of circular statistics in plant phenology: A case studies approach. In: Hudson IL, Keatley MR (eds) Phenological research: Methods for environmental and climate change analysis. Springer, Dordrecht, pp 339–359. 10.1007/978-90-481-3335-2_16

[CR22] Nunes YRF, Souza CS, de Azevedo IFP et al (2022) Vegetation structure and edaphic factors in veredas reflect different conservation status in these threatened areas. For Ecosyst 9:100036. 10.1016/j.fecs.2022.100036

[CR23] Oliveira GC, Araújo GM, Barbosa AAA (2009) Florística e zonação de espécies vegetais em veredas no Triângulo Mineiro, Brasil. Rodriguésia 60:1077–1085. 10.1590/2175-7860200960417

[CR24] Oliveira CS, Messeder JVS, Teixido AL, Arantes MRR, Silveira FAO (2021) Vegetative and reproductive phenology in a tropical grassland–savanna–forest gradient. J Veg Sci 32:e12997. 10.1111/jvs.12997

[CR25] Passos LDO, Lopes A, Bijos NR, Munhoz CBR (2025) Predicting climate change impacts on the distribution of vereda wetland plant species in the Brazilian Cerrado. Annals of Botany:mcaf120. 10.1093/aob/mcaf12 10.1093/aob/mcaf120 40491138

[CR26] PIX4D (2020) PIX4Dmapper, version 4.6. PIX4D SA, Switzerland. https://www.pix4d.com/product/pix4dmapper. Accessed 23 Oct 2024

[CR27] QGIS Development Team (2024) QGIS geographic information system. Open Source Geospatial Foundation Project. https://qgis.org https://qgis.org

[CR28] Ribeiro JF, Walter BMT (2008) As principais fitofisionomias do bioma Cerrado. In: Sano SM, Almeida SP de, Ribeiro JF (eds.). Cerrado: ecologia e flora 1: 151–212. Brasília, DF: Embrapa Informação Tecnológica & Embrapa Cerrados

[CR29] Ribeiro JWF, Pilon NAL, Rossato DR, Durigan G, Kolb RM (2020) The distinct roles of water table depth and soil properties in controlling alternative woodland-grassland states in the Cerrado. Oecologia 195:641–653. 10.1007/s00442-021-04869-z 10.1007/s00442-021-04869-z 33619596

[CR30] Sabino SML, Cassino RF, Gomes MOS, Sant’Anna EME, Rocha Augustin CHR, de Oliveira DA (2021) Late Holocene in central Brazil: vegetation changes and humidity variability in a tropical wetland. J Quat Sci 36:1028–1039. 10.1002/jqs.3351

[CR12] dos Santos GL, Beutler SJ, da Silva CG et al (2025) Consequences of anthropization in the veredas environments in the Brazilian Cerrado. Environ Dev 53:101116. 10.1016/j.envdev.2025.101116

[CR31] Santos RT, Alcantara DMC, Baronio GJ, Moreira SN, Romão DIS, Aoki C, Sigrist MR, Nunes YRF, Souza CS (2025) Continuous flowering of different strata enables resource stability in a tropical Vereda wetland. Wetlands 45:66. 10.1007/s13157-025-01948-2

[CR32] Schreiner-McGraw AP, Vivoni ER, Ajami H, Sala OE, Throop HL, Peters DP (2020) Woody plant encroachment has a larger impact than climate change on dryland water budgets. Sci Rep 10:8112. 10.1038/s41598-020-65094-x 32415221 10.1038/s41598-020-65094-x PMC7229153

[CR33] Silva FHB, Arieira J, Parolin P, Cunha CN, Junk WJ (2016) Floodplain forests of the Iberian Peninsula: vegetation classification and climatic features. Appl Veg Sci 19:391–400. 10.1111/avsc.12219

[CR34] Terasaki Hart DE, Bùi T-N, Di Maggio L, Wang IJ (2025) Global phenology maps reveal the drivers and effects of seasonal asynchrony. Nature 645:133–140. 10.1038/s41586-025-09410-3 40866701 10.1038/s41586-025-09410-3 PMC12408380

[CR35] Trindade VL, Ferreira MC, Costa LS et al (2024) The effect of woody encroachment on taxonomic and functional diversity and soil properties in Cerrado wetlands. Flora 152524. 10.1016/j.flora.2024.152524

[CR36] Vieira LT, Azevedo TN, Castro AA, Matins FR (2022). Reviewing the Cerrado's limits, flora distribution patterns, and conservation status for policy decisions. Land Use Policy 115: 106038. 10.1016/j.landusepol.2022.106038

